# Towards a Low-Cost Mobile Subcutaneous Vein Detection Solution Using Near-Infrared Spectroscopy

**DOI:** 10.1155/2014/365902

**Published:** 2014-04-30

**Authors:** Simon Juric, Vojko Flis, Matjaz Debevc, Andreas Holzinger, Borut Zalik

**Affiliations:** ^1^Advanced ICT Research Group (AIRG), Farmadent Pharm., 2000 Maribor, Slovenia; ^2^Laboratory of Geometric Modelling and Multimedia Algorithms, Faculty of Electrical Engineering and Computer Science, University of Maribor, 2000 Maribor, Slovenia; ^3^Department of Vascular Surgery, University Medical Centre Maribor, 2000 Maribor, Slovenia; ^4^Institute for Media Communication, Faculty of Electrical Engineering and Computer Science, University of Maribor, 2000 Maribor, Slovenia; ^5^Institute for Medical Informatics, Statistics and Documentation, Medical University of Graz, Auenbruggerplatz 2, 8036 Graz, Austria; ^6^Institute of Information Systems and Computer Media, Graz University of Technology, Inffeldgasse 16c, 8010 Graz, Austria

## Abstract

Excessive venipunctures are both time- and resource-consuming events, which cause anxiety, pain, and distress in patients, or can lead to severe harmful injuries. We propose a low-cost mobile health solution for subcutaneous vein detection using near-infrared spectroscopy, along with an assessment of the current state of the art in this field. The first objective of this study was to get a deeper overview of the research topic, through the initial team discussions and a detailed literature review (using both academic and grey literature). The second objective, that is, identifying the commercial systems employing near-infrared spectroscopy, was conducted using the PubMed database. The goal of the third objective was to identify and evaluate (using the IEEE Xplore database) the research efforts in the field of low-cost near-infrared imaging in general, as a basis for the conceptual model of the upcoming prototype. Although the reviewed commercial devices have demonstrated usefulness and value for peripheral veins visualization, other evaluated clinical outcomes are less conclusive. Previous studies regarding low-cost near-infrared systems demonstrated the general feasibility of developing cost-effective vein detection systems; however, their limitations are restricting their applicability to clinical practice. Finally, based on the current findings, we outline the future research direction.

## 1. Introduction

This study outlines necessary steps towards developing a low-cost mobile health solution for subcutaneous vein detection using near-infrared (NIR) spectroscopy [1–3]. The general purpose of this study was to gain a multidimensional understanding of previous works carried out in the field of NIR spectroscopy for the role of vein visualization [4–8]. Our aim was to conceptualize (and later develop) an educational mobile medical application to primarily help to improve decision-making skills of healthcare students (i.e., both nursing and medical) in venipuncture, although the application could also be applied to other purposes (e.g., hospital care and other point-of-care settings). Secondly, we wished to investigate if the available NIR spectroscopy devices were used also for other nonclinical purposes (e.g., as a part of healthcare educational programs). Lastly, our multidisciplinary group wished to ascertain if there has been any precedent in using a standard mobile device for the purpose of vein visualization and to evaluate their scope and findings.

Before proceeding, it is important to define the terminology and illustrate the background of the research used throughout this paper.

Venipuncture is an everyday procedure in healthcare settings. The prevalence of a peripheral venous access line among patients admitted to hospital wards is as high as 90% or more depending on the diagnosis and acuity of the patient as well as the hospital area the patient is in [9]. Although peripheral veins are often accessed with only one needle insertion, in a substantial number of patients it is necessary to practice between 2 and 11 attempts to gain access to a vein [10]. The main causes for the need of multiple attempts are insufficient venipuncture skills, inadequate care and maintenance [11, 12], or a medical condition termed peripheral difficult intravenous access (DIVA) [13–18].

NIR spectroscopy—a technique that makes use of the near infrared region of the electromagnetic spectrum (from 740 nm to 760 nm)—permits the visualization of veins situated 3–5 mm under the skin, veins that are traditionally used for catheterization or blood draw, and it now represents one of the most promising approaches targeted to improve venipunctures' success rate [1–8].

Mobile health (m-health) is an emerging innovation field that is offering a range of promising solutions geared to improving health management [19–21] with the potential to bring advantages into clinical medicine and healthcare [22]. Particularly, the use of smartphones offer very promising possibilities for bringing benefits into the medical area [23–25], and recent studies by Payne et al. [26, 27] found a high level of smartphone ownership and usage among medical students and healthcare professionals, who endorse such applications to support their education and clinical practice and report positive support of productivity and clinical decision making. Moreover, Davies et al. [28] report on the enormous possibilities of such devices for medical education and they have developed a model for mobile learning in the clinical setting that shows how different theories contribute to its use taking into account positive and negative contextual factors.

It was forecasted that there will probably be more than 500 million m-health users in 2015 [29]. Because of the characteristics and functionalities of its products, m-health is gradually redefining healthcare best practices. M-health entails the use of mobile devices in combination with new information and communication technologies and accessories. These devices have given rise to new possibilities in order to overcome obstacles, drive down costs, redesign care-paths and processes, augment the level of patient safety, and improve significantly the quality of care in diverse care settings, including hospital wards, emergency rooms, and homes [30–37].

Even as it was initially a set of relatively simple mobile applications for reminders [38], appointments [39], nutrition counseling [40], general pharmacy practice [41], and self-management of chronic diseases [42], it is nowadays progressing towards a new generation of applications tailored to deal with a broad scope of clinical problems. For example, according to a study performed by Haddock et al. [43], the researchers were able to capture excellent, high-quality fundus (retinal) images in both children under anesthesia and adults awake, by means of the usage of a smartphone along with instruments that are readily available in a standard ophthalmic practice. Rappaport et al. [44] evaluated the reliability and acceptability of an otoscope attachment for a hand-held smartphone, which was able to accurately (in comparison with a conventional otoscope) diagnose ear infections, such as acute otitis media (AOM), in children. Similarly, Lau et al. [45] demonstrated a novel technology to prevent stroke using an iPhone ECG application to detect silent atrial fibrillation, the most common cardiac arrhythmia. For this sort of applications, a new term was introduced by the United States Food and Drug Administration (FDA): mobile medical applications (apps). Mobile medical apps are medical devices that are mobile apps, meet the definition of a medical device, and are, in their turn, an accessory to a regulated medical device or transform a mobile platform into a regulated medical device [46]. Breitwieser et al. [47] report on a smartphone application for viewing biosignals on mobile devices, and Peischl et al. [48] report on the success factors for mobile data acquisition in healthcare.

The main purpose of this study was to gain a complete knowledge, which was needed to define the core concept of the application, as the next step towards converting a standard mobile device (i.e., a tablet or a smartphone) into a low-cost and efficient vein visualization and localization prototype. Therefore, the main objectives of this study were as follows.

The first objective was to get a deeper overview of the problems (i.e., venipuncture and peripheral difficult venous access) and to assess the techniques based on NIR spectroscopy. The second objective was to identify the commercial systems employing NIR spectroscopy, to understand their composition, their effectiveness, and the relevance of clinical studies that they relied on, in addition to appraising their clinical outcomes and limitations. The third objective was to review all about the research efforts in the field of low-cost NIR spectroscopy, especially, its strengths, weaknesses, and technical features.

## 2. Methods

### 2.1. Research Group

It is well established that when developing a medical device, it is important to involve and consider different perspectives (e.g., manufacturers', health professionals', and users') at every stage of the development lifecycle (starting from the early stage of defining the core concept), to optimize clinical usability, improve patient safety, reduce development time and costs, and achieve higher levels of patient and user satisfaction, which all result in a higher quality end product [49–52]. However, for a majority of mobile medical apps (available through official application stores for the major smartphone platforms; see a recent work here [53]), there is a lack of evidence on their clinical evaluated effectiveness, utility, and safety [54–58]. Furthermore, most of them do not even mention any sort of health professionals' or accuracy assessments during their development, which questions their quality in delivering health-related interventions. For example, in a study performed by Bender et al. [58], where the researchers reviewed and analyzed cancer-related smartphone apps, they were not able to identify any sort of evaluations of these apps.

In this sense, a multidisciplinary research group (whereby researchers and developers teamed up with clinicians and other health professionals) was established, with the aim to, guided by a user-centered design approach and an engineering and usability process framework [49, 50, 59], evaluate the design (and a later prototype) through multiple iterations from the clinical and technical points of view.

In order to attain the main objectives in this study, the research methodology was comprised of three steps and was conducted between September and December 2013 (revised in February 2014).

### 2.2. Phase 1

As the initial step, the purpose of this phase was to gain a deeper overview about the general challenges of peripheral vein access, especially concerning the background, scope and the improvement strategies related to the handling of venipuncture. After the initial team discussions, enriched and guided by the health professionals, we carried out a detailed literature review (using academic and grey literature) about a group of interrelated terms, leading to a comprehensive knowledge about the research topic.

### 2.3. Phase 2

**Figure 1:**
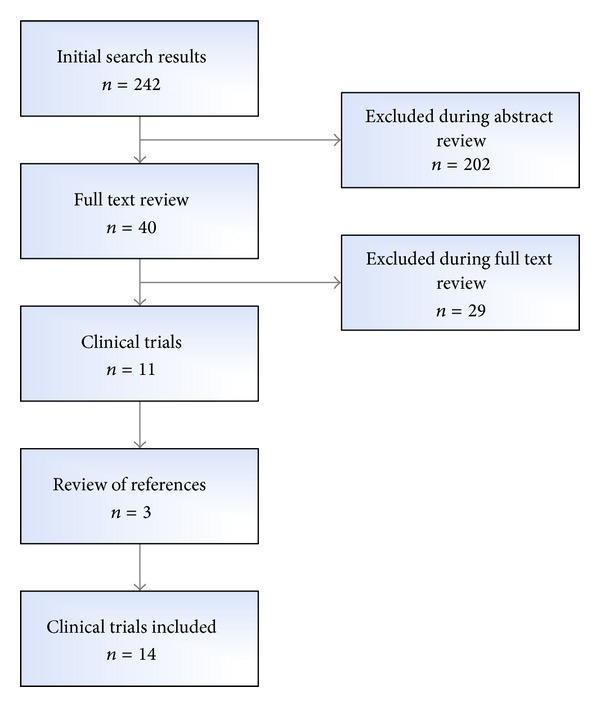
PubMed search strategy and literature selection process.

In order to address the second objective (i.e., to identify the commercial systems employing NIR spectroscopy), a systemic search was conducted using the PubMed database maintained by the National Library of Medicine at the National Institutes of Health and other sources. A search strategy (the flow can be seen in Additional File 1 in Supplementary Material S1 available online at http://dx.doi.org/10.1155/2014/365902) was constructed to sort out the published papers that tested NIR imaging to help visualize the vein pattern to choose the most appropriate skin point for venipuncture or cannulation. After collecting the abstracts that were returned using the search strategy, a manual review was performed to select relevant articles. Full-length articles were obtained for the most relevant abstracts. Clinical studies and efficacy studies were separated and other articles (i.e., systematic reviews, reviews, technical papers, etc.) were used as the reference base. The bibliography from the full-length articles was used to identify any additional journal articles that were not identified during the Boolean search. A flowchart that describes the selection process is shown in Figure 1.

### 2.4. Phase 3

One of the aims of m-health is to provide cost-effective alternatives in the form of mobile medical apps, especially in the field of diagnostics. Guided by this aim, the goal of our third objective was to identify and evaluate the research efforts in the field of low-cost NIR imaging in general. By the term low-cost research we are referring to studies (e.g., feasibility and formative studies) which successfully experimented with developing any sort of NIR imaging or acquisition systems from low-cost components. As our next step is to define a concept of transforming a standard mobile device into a low-cost and efficient vein visualization device, the related research findings could have a valuable impact on the design of the model and the architecture of the forthcoming device.

**Figure 2:**
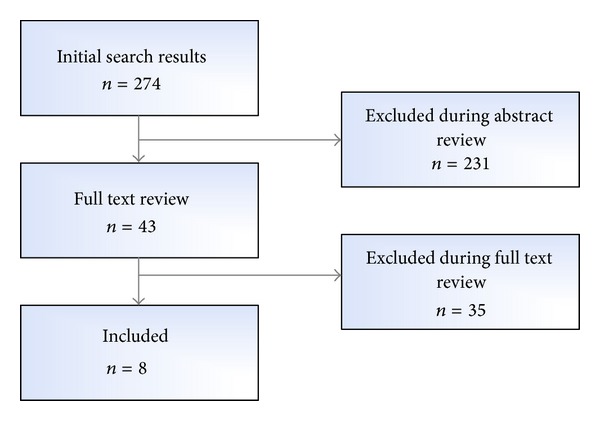
IEEE Xplore database search strategy and literature selection process.

In this sense, a systematic search was conducted using the IEEE Xplore database provided by the world's largest professional association for the advancement of technology, Institute of Electrical and Electronics Engineers (IEEE). The search strategy (included in the Additional File 1 in the Supplementary Material S1) was used to identify papers covering the design or evaluation of NIR-related systems for multiple purposes, focused on their technical and usability features (e.g., design architectures, components, and other formative experiences). A flowchart that describes the selection process is shown in Figure 2.

## 3. Results 

### 3.1. Phase 1: Peripheral Vein Access and Venipuncture

Venipuncture, the process of obtaining intravenous (IV) access, is an everyday invasive procedure in medical settings and there are more than 1 billion venipuncture related procedures (i.e., blood draws, peripheral catheter insertions, and other IV therapies) performed per year [60]. It is well documented that excessive venipunctures are a significant challenge in today's healthcare institutions [9]. Especially in infants and children, recurrent attempts to insert a needle to gain access to a vein elicit anxiety, pain, and distress [10, 11] and elevate the risk of damaging the veins causing infiltration of the surrounding area and the subsequent possibility of a catheter-related, hospital-acquired bloodstream infection [15–17].

Children are probably the most commonly studied population as their experience is most traumatic. Children can be difficult to catheterize due to lack of cooperation, decreased amount of subcutaneous fat, and smaller veins [61]. An average of 2.35 attempts is necessary to insert a peripheral IV catheter (range 1–10.5) in a child [3]. Less than half is inserted in the first attempt, about 2/3 is inserted after two tries, and in 5% a catheter is not inserted. There is a lower success rate in infants. 74% of hospitalized children report that peripheral venous catheter placement is the cause of their worst pain [62].

Due to the fact that alternative routes for the administration of drugs, fluids, or blood are not always feasible and that multiple attempts of needle insertion are time- and resource-consuming (e.g., catheters, needles) events, it was necessary to develop other approaches geared to improve venipunctures' success rate, especially in difficult cases [9]. In addition to the standard technique of visualization and palpation, there are four main options to complement it, as follows: (1) manual procedures with the support of local chemicals, but they have limitations, above all, in children and people with dark skin [17], (2) ultrasound-based procedures, but they require the intervention of trained staff and expensive equipment [18], (3) the use of additional sources of light in a darkened room, which can raise the risk of skin burns [63], and, finally, (4) the visualization of the vein system through NIR spectroscopy [2, 3].

From a high-level architecture (HLA) perspective, an NIR spectroscope comprises two elements: (1) an NIR light source and (2) an NIR-sensitive camera that is capable of capturing the surface illuminated by the light source (i.e., the NIR image), which is then further processed and displayed for clinical usage [1–3].

This imaging technique allows a clear visualization of the subcutaneous vein pattern as a consequence of the different optical properties of the circulating human blood and tissues. The difference lies in the fact that veins are rich in deoxygenated hemoglobin, a molecule that almost completely absorbs the NIR light, in contrast to arteries that, instead, contain oxygenated hemoglobin [64, 65]. As a result, the vein pattern appears highlighted in comparison with the surrounding tissue, which is the striking quality of any NIR imaging device.

### 3.2. Phase 2: Commercial Systems Employing NIR Spectroscopy

Based on the systematic search, we identified three devices that use NIR spectroscopy to facilitate peripheral IV catheter insertion or blood draw and they were subject to several clinical evaluations. They were the VeinViewer [1, 66] (Christie Medical Holdings, Memphis, TN, USA), VascuLuminator [67] (De Koningh Medical Systems, Arnhem, NL), and AccuVein [68] (AccuVein LLC, Cold Spring Harbor, NY, USA). The VeinViewer was approved for use by the FDA in 2005 [66]. The AccuVein (AV300) was approved by the FDA in 2009 [69] and it is regarded as the first hand-held NIR device.

Although the three devices differ in the design and implementation and use different components, they all comply with the same aforementioned core HLA design.

The VeinViewer, through employment of an NIR light-emitting diode and a laser projector, analyzes the skin image and projects the vein's image back on the skin [70], where the veins appear as black lines on a green background. The AccuVein device operates in a similar manner, albeit projecting black lines on a red background [3]. The VascuLuminator, which consists of a NIR light source placed underneath the puncture site and a camera that displays the puncture site, projects the image to a screen where the veins appear black on a light background [3].

The systematic search yielded 14 clinical studies that assessed the aforementioned devices as an assistance aid in vein identification, venipuncture, and cannulation (Table 1).

Perry et al. [73], in a study of peripheral IV catheter insertion in emergency room (ER) patients (0–20 years old), reported that even though there was no improvement in the first attempt success rate associated with the use of VeinViewer, 90% of the surveyed pediatric ER nurses expressed that the device was helpful in difficult access patients.

Chapman et al. [72] evaluated the efficacy of VeinViewer in a group of patients (0–17 years old) compared to that of standard care. Their results showed that there was no improvement in time to peripheral IV catheter placement, number of attempts, or pain scores in cases associated with use of the device. However, a subgroup analysis of children (0–2 years old) demonstrated a benefit in peripheral IV catheter insertion time (121 seconds versus 167 seconds) and a lower first failure rate (ratio of geometric means: 1.39; CI: 1.29–1.82). The experienced ER pediatric nurses most frequently (73.5%) defined the use of the NIR device as easy. This helpfulness did not change with the level of difficulty of the catheter insertion.

Another clinical trial conducted by Kim et al. [75] in patients (1 month–16 years old) evaluated IV cannulation performed by hospital nurses using the VeinViewer versus the standard technique. There was no difference in the overall first attempt success rate. But, given that some patients were technically challenging for IV catheter insertion, as described by the difficult intravenous access (DIVA) score [13–18], a subgroup analysis focused on the difficult access children revealed a smaller first attempt failure rate (58.3 versus 25.0%) using an NIR device [61].

Phipps et al. [66], in another study that assessed peripheral intravenous catheter insertion carried out by a neonatal nursing team in preterm and term neonates, reported that patients of similar gestational age had a higher success of IV placement associated with the use of the VeinViewer (adjusted odds ratio: 3.05).

In the study conducted by Kadddoum et al. [68] performed by experienced pediatric anesthesiologists in the operating room (OR) in patients (0–17.1 years old), the efficacy of AccuVein was compared to that of standard care, but this time no difference was seen in the first attempt success rate, number of skin punctures, or the time to successful cannulation of the two groups.

In another clinical trial of peripheral IV catheter insertion in pediatric intensive care unit (ICU) patients (3 months–17 years old), Sun et al. [78] found that the NIR device was associated with a shorter time to identify an appropriate vein (126.37 versus 383.61 seconds) and a fewer number of attempts required (median: 1 versus 2) compared to the standard technique. Also, the time for IV placement was shorter in the NIR group (186.2 versus 497.2 seconds), a relevant factor in critically ill patients.

In a clinical trial of children (0–18 years old), in whom experienced pediatric anesthesiologists, nurse anesthetists, trainee anesthesiologists, and trainee nurse anesthetists compared intravenous cannulation using a VascuLuminator device with the standard technique before elective surgery, Cuper et al. [77] reported that there was no improvement in the observed success rate or time to success with the NIR device, though the authors suggested that the selection of patients could have been inappropriate.

In the study conducted by de Graaff et al. [3], the team compared the use of VeinViewer, AccuVein, VascuLuminator, and a control group in peripheral vein catheterization before elective surgery in a group of patients (0–18 years old). The results showed that suitable veins were easily identified using the VeinViewer (95.3% of patients) and AccuVein (94.1% of patients), more than using the VascuLuminator (89.1% of patients). However, there was no significant difference in the rate of successful IV placement in the four groups, even though this study was performed by experienced pediatric anesthesiologists, nurse anesthetists, trainee anesthesiologists, and trainee nurse anesthetists.

According to van der Woude et al. [79], in a recent study performed by anesthesiologists in dark skinned children (0–15 years old), in which the authors compared the use of the VascuLuminator device with the standard technique prior to elective surgery, no benefit was seen in success at first attempt or in time to successful cannulation.

In another clinical trial conducted by Hess [70], VeinViewer was used by nurses to perform a series of peripheral IV catheter insertions in a group of patients (newborn to 17 years old) during patient hospitalization. In this case, the use of the NIR device was associated with a 31% improvement in first stick insertion success, a 35% decrease in the number of attempts, and a 39% decrease in time required for IV insertion compared to historical controls at the same unit using standard care.

Finally, in a study performed by trained phlebotomists led by Cuper et al. [71], VascuLuminator was used to facilitate blood draws in children (0 to 6 years). In these children, NIR imaging was associated with an improved failure rate (10/80 versus 1/45) and decreased blood draw time, compared to historical controls.

The results and characteristics of all these studies that employed NIR spectroscopy have been summarized in Table 2.

### 3.3. Phase 3: Low-Cost NIR Imaging Prototypes

Based on the systematic search and the previous engineering experiences (from the researchers and developers participating in our research group), we identified 8 formative or feasibility studies that offered us valuable resources in defining the concept (model and architecture) of transforming a standard mobile device into a low-cost and efficient vein visualization device.

In the first identified study, performed by Crisan et al. [5], the researchers demonstrated an experimental low-cost multipurpose infrared (IR) biometric system, comprised of a modified regular Universal Serial Bus (USB) camera (which is NIR-sensitive), a set of NIR light-emitting diodes (LED) as a lighting (illumination) source, and a desktop computer. In this respect, it is worth knowing that a regular camera is equipped with a NIR cut filter; that is, it cuts the wavelength above 720 nm and, therefore, disables capturing NIR images, so that it needs to be removed and replaced with an NIR pass-through filter, which cuts visible light up to 720 nm and passes through light with wavelength above 720 nm [6, 80]. The proposed architecture enabled the researchers to further enhance the acquired NIR image by applying a series of digital signal processing (DSP) algorithms, running on the computer. In a follow-up study [6], the researchers further elaborated on the qualitative aspects and implementation considerations regarding the prototype usage as a medical aid or as a biometric scanner. Similar architecture and approach was proposed in a later study performed by Mansoor et al. [4], where, interestingly, besides the cost-effectiveness, the authors emphasized the portability of the device, yet the architecture still used a computer as the core component.

Zhao et al. [81] describe a prototype, which additionally enables the usage of two separate sets of lighting sources (using NIR LEDs), in order to illuminate the surface of the two sides of the hand and, therefore, extends the NIR penetration deeper into the tissue in order to obtain a better contrast in the acquired NIR image. The authors also presented a denoising algorithm as further enhancement of the acquired image.

A study performed by Chakravorty et al. [82] successfully applied and evaluated the model proposed by Crisan et al. [5] on an ARM-based single-board computer (SBC) as a replacement for a regular computer and, therefore, reduced the size of the proposed prototype. Gayathri et al. [83] performed a similar work in applying the model to an Embedded Linux Platform and evaluated the device as a low-cost hand vein authentication system. With the same aim (i.e., authentication), Liu and Song [84] proposed a novel finger-vein NIR-based recognition system, which groups all the required components (i.e., illumination, image acquiring, and DSP) into a single hardware module. Furthermore, the proposed architecture enabled low-power consumption with a minor impact on the efficacy.

The aforementioned studies are valuable examples to set the ground to demonstrate the general feasibility of developing low-cost vein detection systems, but they also present a series of limitations (e.g., architecture, experimental set-up, and limited assessments) impacting their applicability outside the research settings (i.e., to clinical practice). Furthermore, and with respect to the main aim of this study, although some of the described prototypes underwent preliminary testing on subjects of different gender, age, complexion, and body type, none of them was evaluated for efficacy or general suitability as a venipuncture-related vein visualization assistance aid. The latter, together with the aforementioned limitations, will be addressed as our main design objective in the upcoming conceptualization and development phases.

We also identified one brief conference proceedings paper by Nundy and Sanyal [85] that shows some similarities with our main objective (i.e., vein visualization on a standard mobile device). This paper proposes a design in the form of a vein detection system using an array of NIR LEDs and a built-in camera of a mobile phone. Although this research aims at the same direction, but with a limited scope, we argue that the proposed and briefly described architecture could be of value inasmuch as it would be applicable to most of the mobile devices on the market (on this point, we will further elaborate in Section 4.3).

## 4. Discussion 

### 4.1. Principal Findings

NIR imaging is the newest technology to become available as a guidance tool to facilitate vein identification, especially beneficial for high risk populations (Table 3) where peripheral IV catheter insertion or blood draw are difficult and result in excessive attempts to gain access to a vein (i.e., venipunctures).

Several NIR imaging devices are already available for use and have been recently assessed in different clinical settings for a variety of purposes. Other less bulky and more portable commercial devices are also under development, for example, a head-mounted system Veinsite [8] (VueTek, Grey, ME, USA) and similar device Evena Eyes-On Glasses [86] (Evena Medical, Los Altos, CA, USA), which also provides multispectral imaging capability (besides NIR).

Although the reviewed devices have demonstrated usefulness and value for peripheral veins visualization, other evaluated factors were less definite in order to preclude any general conclusions about their overall efficacy regarding the observed clinical outcomes (e.g., improving the success rate of venipuncture at first attempt or decreasing the procedure time for peripherally IV insertion of catheters). While some of the reviewed studies demonstrated no benefit with technology in first attempt insertion rate [68, 73, 74], a benefit in shorter time to identify an appropriate vein [70, 71, 75], a fewer number of attempts were required [70, 78], and shorter time for IV placement was documented [70–72, 78].

We believe that the reasons for these inconclusive findings are mostly originating in the fact that the employment of NIR imaging technology for this purpose is a new approach as the current studies started to appear in scientific journals from 2010 and are mostly based on limited clinical settings. Furthermore, we speculate that the availability of these commercial devices (considering the related costs as the major factor, followed by availability in different countries and the training time needed) is another obstacle towards more comprehensive studies on larger samples. This also corresponds to a recent study by Lamperti and Pittiruti [60], where they identified bias shared in most of the related efficacy studies of these devices, namely. (1) exclusive focus on pediatric population, (2) sampling of limited number of cases, (3) difficult or inappropriate randomization, (4) poor standardization of the procedure, (5) wrong definition of the clinical endpoints, (5) lack of consideration of the need of a proper training with the device, and (6) little or no consideration of the level of experience of the operator performing the procedure [60]. The authors also brought into consideration three main unresolved issues, whereby these devices are still far away from a wider application in clinical practice (hospitals), as follows: (1) training needed, as most of the studies do not reveal how many procedures or time the operators needed in order to be considered proficient with their usage, (2) cost-effectiveness analysis in comparing the raw cost of the device (several thousand dollars) and the needed training versus the time-saving and clinical benefits, and (3) other technical concerns.

### 4.2. Potential for Other Nonclinical Usage

Furthermore, from the papers reviewed here and the previous knowledge inside our research group, there is no evidence to date of any use or assessments of commercial NIR imaging systems outside clinical research settings. In our multistage research, we aim to assess their efficacy as an educational aid where we think they could play a vital role in the development of necessary skills (of medical as well as nursing students) in clinical decision making in a variety of areas (especially, in venipuncture), with the main purpose of providing safer patient care and preventing excessive venipuncture-related medication errors in the future (once these students start working as new healthcare professionals). Therefore, we believe that the multipurpose potential of these devices has not yet been investigated thoroughly, which is the main research objective of our upcoming low-cost solution. We also believe that our solution, based on a standard mobile device and its lower cost (in contrast to the available or future commercial devices), could increase the availability of such imaging devices and therefore expand their reach also into healthcare education, among other areas.

Today's nursing education methods and content are undergoing a rapid redefinition with an emphasis on innovation management and the usage of emerging technologies as part of the nursing curricula [87–91]. With the aim of helping students to develop enhanced skills and provide them instant access to best practice information (that can be applied in both simulated and clinical practice settings), and as a means of transferring learning into clinical practice, there are several successful examples of integrating technology into healthcare education ranging from smartphones and tablets-based apps to high-tech programmable simulators [92–95].

Mobile technologies are an essential part in this educational transformation, inasmuch as their innovative features and widespread availability makes them very valuable educational tools. Besides, their ability to provide instant and more frequently updated evidence-based practice information (through mobile apps or interned-based resources) along with the latest advances in m-health (in terms of transforming a mobile platform into a regulated medical device-mobile medical apps) will make mobile devices an even better platform to analyze and synthesize critical health-related information, especially in the field of diagnostics. Similarly, given that today we are able to install and use several m-health apps (available on several mobile marketplaces), we will be able to purchase (or build) a specific hardware accessory and, coupling with an accompanying software-based mobile app, transform a standard mobile device (e.g., smartphone) into a low-cost and effective diagnostic tool. The latter and the ability of quickly switching the hardware accessory and turning the device into a different medical aid (for a fraction of the cost comparing to the high-end commercial devices) suggest a viable potential for their usage in the current and the upcoming technology-oriented educational framework.

In this sense, our main purpose is to develop an education-focused mobile medical application to help improve the decision-making skills of healthcare students (i.e., both nursing and medical practitioners) in venipuncture, although the application could also be later applied to other purposes. Moreover, existing (printed or electronic) practice guidelines, handbooks, norms, and policies can be now complemented by several recent technology innovations targeted to enhance the learning experience of students regarding procedures such as venipuncture or phlebotomy in general. As an example, Vidal et al. [96] compared the effectiveness of virtual reality simulators on developing phlebotomy skills of nursing students with the effectiveness of traditional methods of teaching. Their findings, through a quasi-experimental study, demonstrated that students, who were exposed to the virtual reality simulator, performed better (based on the observed metrics: pain factor, hematoma formation, and number of attempts to insert the needle) in the actual phlebotomy on a live client than the control group. In a similar sense, our hypothesis is that our solution could offer a viable supplement to the existing traditional methods of teaching and consequently enhance the quality of training of future nurses in their care practice.

### 4.3. Conceptual Model

**Figure 3:**
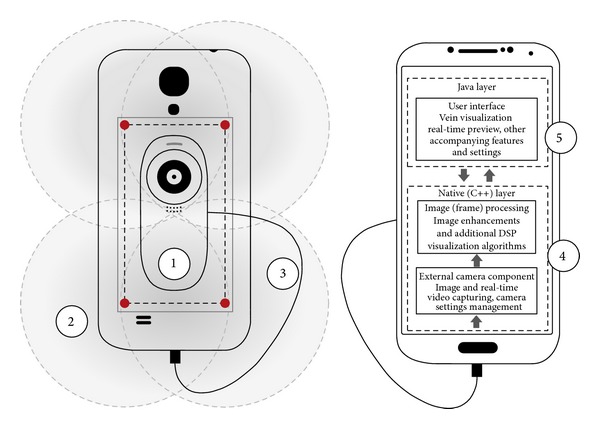
High level conceptual model and architecture of the prototype, embedded in a wireframe of one of the target devices (smartphone). (1) Attachment which comprises a standard (converted to be NIR sensitive) USB camera and 4 NIR LEDs for the illumination of the target area (e.g., hand). (2) Illustration of 1 NIR LED illumination distribution, with the most luminous intensity around the center. (3) Micro-USB connection cable (data transmission and power supply for the attachment). (4) Software libraries (camera usage and management, image processing) written in the native Android layer (for performance optimizations). (5) User interface software components. Both (4) and (5) will be developed as a single Android-based mobile application applicable to mobile devices running Android OS version 4.0.3 (ICS) or above.

Based on the current findings and several identified advantages of developing a low-cost m-health application for subcutaneous vein detection using NIR spectroscopy, our research group has already began experimenting towards that direction. The current conceptual model (Figure 3), which will later evolve into the prototype architecture, is based on the following three elements, namely, (1) a general high-level architecture of an NIR spectroscope (i.e., NIR-sensitive camera and illumination source), (2) the previous experimental research findings and identified limitations (see Section 3.3) in low-cost NIR spectroscopy, and (3) the identified features of commercial devices (e.g., additional augmented vein visualization processing).

In this sense, the current model (and the upcoming application) extends previous low-cost research and proposes a standard mobile device-based practical end result, in sync with that envisioned by Crisan et al. [5] (one of the first low-cost related studies) in which they concluded that the final result should be a self-contained low-power device capable of accurately detecting different vein patterns. Based on this, we postulated that the following basic design objectives should be based on (1) taking advantage of the functionalities and the hardware of the device as much as possible, (2) using only low-cost hardware which should have to be easily installable, (3) being powered only by the device itself, (4) being very simple to use, and (5) being capable of visualizing the veins in real time as clearly as the commercial devices. In this respect, the whole cost of the final solution (hardware and software) could not exceed US$50.00 (excluding the mobile device itself).

After evaluating other alternatives, we chose Android (Google, USA) technology as the appropriate platform for the initial prototype, inasmuch as it offers an open platform and a wide spectrum of future development possibilities. Furthermore, it allows us to take into account the potential usage of the upcoming Google Glass (Google, USA) platform, as one of the design objectives during this product development phase.

In accordance with our main objectives, our team began a sequence of feasibility studies in order to identify how to apply the abovementioned model to the target platform, as the initial phase in our upcoming research to develop a new prototype. After a series of experiments and analyses of the built-in cameras in a variety of mobile devices, we confirmed our initial hypothesis that all of them have an NIR cut filter present (i.e., they cut the wavelength above 720 nm) and, therefore, this disables capturing NIR images (the basic requirement of an NIR spectroscope). The main purpose of this filter is to enable normal photography without any interference caused by the NIR-light spectrum and is present in most of the everyday camera-based devices. Although the filter could be removed, the process (on a mobile device with a built-in camera) is complex and almost irreversible. Based on this, we argue that the model proposed by Nundy and Sanyal [85] would be applicable to most of the mobile devices on the market. Though the authors, in their briefly described architecture of the model, do not disclose which devices they are targeting, we can speculate (based on our knowledge about the mobile devices market) that their model could only be applicable to older and rare low-end feature phones [97, 98], which do not have a NIR cut filter present. Furthermore, the sufficiency of such a solution, based on the limitations of these devices (e.g., small screens, low-quality photos, and limited programmability support) and the ergonomics (as they do not reveal how the attachment will be powered), is also questionable. Nevertheless, as their research aims at the same direction (although with a limited scope as compared to ours), a comparative study (evaluating the efficacy, scope, and usability) would be valuable, if their model gets developed in the future.

### 4.4. Future Work

**Figure 4:**
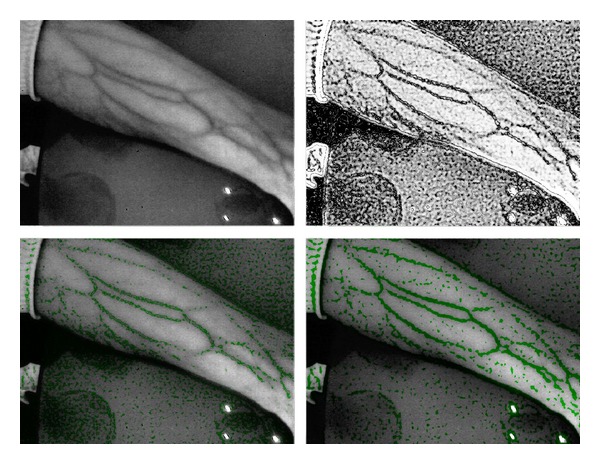
Current result of the ongoing feasibility studies applying the proposed model to the target platform (standard mobile device). The first image shows the acquired image (converted to grayscale) using the initial version of the prototype (based on Figure 3). Subsequent images present the interim results of applying digital signal processing strategies in order to enhance and additionally visualize the vein pattern.

As aforementioned, our team already began a set of feasibilities studies regarding the applicability of the basic components of an NIR spectroscope to the target platform. As the usage of the built-in camera was not an option and special NIR-sensitive cameras are expensive, we had to revert to existing low-cost experiments by using an external USB camera (for the initial prototype) which was already proven as a suitable solution [4–6]. In this stage, we are evaluating how to connect the camera to a mobile device and to make them function as a coherent system, where we already have some successful results. In regard to the second important component (NIR illumination), we are evaluating different NIR-light sources with a focus on a set of low-energy NIR LEDs, different combination of placements, and experimenting how to efficiently integrate them into the upcoming architecture. As every identified commercial system for NIR spectroscopy uses at least some digital signal processing strategies, in order to process the acquired image (and thereby enhance or additionally visualize the vein patterns) before it is displayed for clinical usage, we are experimenting and identifying how to apply these processing strategies into the architecture.  Figure 4 shows the interim feasibility results achieved in this phase.

After the feasibility studies are concluded, the next phase will involve a rapid iterative development process, where the evolving prototype will be evaluated from the clinical and technical perspectives, based on which a list of tasks for subsequent improvements for the next iteration will be defined. After the prototype is developed, the third phase will evaluate its usability, safety, and accuracy on a limited spectrum of clinical settings. The fourth phase will, through a noninvasive observational feasibility study as an educational aid, evaluate the prototype suitability in visualizing and localizing the veins by comparing its efficacy and the nursing students' skills. At a later stage (planned between Q4 2014 and Q1 2015), the efficacy of the device will be assessed during multiple invasive procedures (i.e., blood withdrawal or IV cannulation) in a randomized clinical trial.

## 5. Conclusions 

New technological innovations are becoming available that deliver practical, reliable, and low-cost solutions to the healthcare sector. These m-health applications promise to change the way we care for patients, educate practitioners, and manage health organizations. Both hospitals and primary care centers will increasingly rely on these new devices to comply with improved standards of quality care. In this sense, this review-based study outlines the first step towards converting a standard mobile device (i.e., a tablet or a smartphone) into a low-cost and effective vein visualization prototype, which will be developed as a result of the introduced multiphase-based research in the upcoming months.

In contrast to other commercial systems that employ NIR technology for the purpose of vein visualization, the end solution (the developed software accompanying other technical details related to the used hardware components) will be freely available under the GNU General Public License.

## Supplementary Material

The supplementary material outlines the query-based search strategies for Phase 2 (PubMed database) and Phase 3 (IEEE Xplore database) as the basis for literature selection process.

## Figures and Tables

**Table 1:** Overview of clinical studies evaluating NIR spectroscopy employment for medical applications.

First author, year	Study design	Subject age	Number of study patients	Procedure	Procedure performed by	Procedure setting	Device used	Benefit to technology
Hess [70] (2010)	Historical control	3 days–17 years	91	Peripheral IV catheter insertion	Nurses	Hospital	VeinViewer	Benefit

Strehle [1] (2010)	Nonrandomized prospective	0–16 years	50	Peripheral IV catheter insertion or blood draw	Consultants, registrars, senior house officers, and nurses	Outpatient	VeinViewer	N/A

Cuper et al. [71] (2011)	Historical control	0–6 years	125	Blood draw	Phlebotomists	Phlebotomy station	VascuLuminator	Benefit

Chapman et al. [72] (2011)	Prospective randomized controlled	0–17 years	323	Peripheral IV catheter insertion	Pediatric ER nurses	Pediatric ER, elective IVs	VeinViewer	Benefit

Perry et al. [73] (2011)	Prospective randomized controlled	0–20 years	123	Peripheral IV catheter insertion	Pediatric ER nurses	Pediatric ER, elective IVs	VeinViewer	No benefit

Cuper et al. [74] (2012)	Nonrandomized prospective	<3 years	77	Arterial line insertion	Experienced pediatric anesthesiologists and nurse anesthetists	Operating room, before cardiothoracic surgery	VascuLuminator	No benefit

Kaddoum et al. [68] (2012)	Prospective randomized controlled	0.18–17.1 years	146	Peripheral IV catheter insertion	Experienced pediatric anesthesiologists	Operating room, elective procedures	AccuVein AV 300	No benefit

Kim et al. [75] (2012)	Prospective randomized controlled	1 month–16 years	111	Peripheral IV catheter insertion	Nurses	Hospital	VeinViewer	Benefit

Peterson et al. [76] (2012)	Nonrandomized prospective	0–19 years	155	Peripheral IV catheter insertion	Nurses	Hospital	VeinViewer	No benefit

Phipps et al. [66] (2012)	Prospective randomized controlled	Preterm and term neonates (23–40 weeks)	115	Peripheral IV catheter insertion	Expert level neonatal nurse practitioners and registered nurse peripheral IV catheter team	Hospital	VeinViewer	Benefit

Cuper et al. [77] (2013)	Cluster randomized	0–18 years	494	Peripheral IV catheter insertion	Experienced pediatric anesthesiologists, nurse anesthetists, and trainees	Operating room, elective procedures	VascuLuminator	No benefit

de Graaff et al. [3] (2013)	Cluster randomized	0–18 years	1383	Peripheral IV catheter insertion	Experienced pediatric anesthesiologists, nurse anesthetists, and trainees	Operating room, elective procedures	VeinViewer, AccuVein AV 300, VascuLuminator	No benefit

Sun et al. [78] (2013)	Prospective randomized controlled	3 months–17 years	60	Peripheral IV catheter insertion	Nurses	Pediatric ICU	VeinViewer	Benefit

van der Woude et al. [79] (2013)	Cluster randomized	0–15 years	88	Peripheral IV catheter insertion	Anesthesiologists	Operating room, elective procedures	VascuLuminator	No benefit

Hess [70] (2010) evaluated patients for 6 months using standard techniques and compared the results to patients treated subsequently with the VeinViewer. Cuper et al. [71] (2011) evaluated patients for 10 weeks using standard techniques and compared the results to patients treated subsequently with the VascuLuminator. *Benefits to technology* were any considered benefits measured in the study populations (described in more detail in Table 2). N/A signifies no analysis performed (feasibility or less rigorous study demonstrating the general usability of the device).

**Table 2:** Summary of Clinical Benefits of NIR spectroscopy employment.

First author, year	Benefit to technology	Group with no significant findings	Subgroup with significant findings	Benefits seen					
				First attempt success	Mean number of attempts	Time of procedure	Less Pain	Overall success	Nurses felt the device was helpful in difficult patients
Hess [70] (2010)	Benefit	N/A	All patients young	X	X	X			

Strehle [1] (2010)	N/A	Used only to identify veins							

Cuper et al. [71] (2011)	Benefit	N/A	All patients young	X		X			

Chapman et al. [72] (2011)	Benefit	All patients	0–2 years			X	X		

Perry et al. [73] (2011)	No benefit	All patients	None						X

Cuper et al. [74] (2012)	No benefit	All patients	None						

Kaddoum et al. [68] (2012)	No benefit	All patients	None						

Kim et al. [75] (2012)	Benefit	All patients	Difficult vein DIVA score >4	X					

Peterson et al. [76] (2012)	No benefit	All patients	No subgroup analyses						

Phipps et al. [66] (2012)	Benefit	All patients very young	Groups matched for gestational age					X	

Cuper et al. [77] (2013)	No Benefit	All patients	No improvement with subgroup analysis						

de Graaff et al. [3] (2013)	No Benefit	All patients	No improvement with subgroup analysis						

Sun et al. [78] 2013	Benefit	All patients	No subgroup analyses		X	X			

van der Woude et al. [79] (2013)	No Benefit	All patients	Children considered to have “difficult” veins	X					

*Benefit to technology* is listed as in Table 1. Overall analyses are described in *Group with no significant findings*. Results of subgroup analyses are presented in *Subgroup with significant findings*. *Benefits Seen* describes the benefits measured in the study. N/A signifies no analysis performed.

**Table 3:** Risk factors for difficult IV insertion [7, 8, 10, 70].

Risk parameter	
Dehydration	
History of difficult access	
Dark skin	
Obesity	
Hypotension	
Peripheral vasoconstriction	
Poor vein quality	
History of previous IV insertions	
Advanced age	
Young age, preterm and term neonates	
Drug abuse	
History of chemotherapy	
Telangiectasia	
Skin rash	
Low skill of the operator	

## Abbreviations

AOM: Acute otitis media
ARM: A family of instruction set architectures for computer processors based on a reduced instruction set computing architecture developed by British company ARM Holdings
DIVA: Difficult intravenous access
DSP: Digital signal processing
ECG: Electrocardiogram
ER: Emergency room
FDA: Food and Drug Administration
GNU: Most widely used free software license
HLA: High-level architecture
ICU: Intensive care unit
IEEE: Institute of Electrical and Electronics Engineers
IV: Intravenous
LED: Light-emitting diodes
NIR: Near-infrared
OP: Operating room
SBC: Single-board computer
USB: Universal serial bus.
