# Supplementary material for: Towards a Low-Cost Mobile Subcutaneous Vein Detection Solution Using Near-Infrared Spectroscopy
Source: ScientificWorldJournal. 2014 Apr 30;2014:365902. doi: 10.1155/2014/365902 (PMC4032719; doi:10.1155/2014/365902)
Supplement: Supplementary file 1 — The supplementary material outlines the query-based search strategies for Phase 2 (PubMed database) and Phase 3 (IEEE Xplore database) as the basis for literature selection process. [file 365902.f1.docx]

1. **Phase 2 – Search Strategy, PubMed database**

Available at: <http://www.ncbi.nlm.nih.gov/pubmed/>

| Search | Query | Items found |
| --- | --- | --- |
| #23 | #7 OR #22 | **242** |
| #22 | #21 AND #17 | 20 |
| #21 | #19 OR #20 | 21 |
| #20 | Optical coherence tomography AND phlebotomy | 1 |
| #19 | infrared AND phlebotomy | 20 |
| #18 | NIR AND #17 | 1 |
| #17 | #15 OR #16 | 24 |
| #16 | #14 AND infrared | 24 |
| #15 | #14 AND near-infrared | 20 |
| #14 | #12 AND #13 | 8617 |
| #13 | venepuncture | 781 |
| #12 | venipuncture | 8022 |
| #11 | (("Spectroscopy, Near-Infrared"[Mesh]) AND venepuncture) | 0 |
| #10 | #8 OR #9 | 11 |
| #9 | (("Spectroscopy, Near-Infrared"[Mesh]) AND venipuncture) | 11 |
| #8 | (("Spectroscopy, Near-Infrared"[Mesh]) AND "Phlebotomy"[Mesh]) | 6 |
| #7 | #5 OR #6 | 224 |
| #6 | #3 AND #4 | 168 |
| #5 | #1 AND #2 | 59 |
| #4 | ((Optical coherence tomography) AND cannulation) | 435 |
| #3 | ((Optical coherence tomography) AND venous) | 637 |
| #2 | ((near-infrared) AND cannulation) | 161 |
| #1 | ((near-infrared) AND venous) | 742 |

1. **Phase 3 – Search Strategy, IEEE Xplore database**

Available at: <http://ieeexplore.ieee.org/>

Advance search mode (full text and metadata), date range from 2005-2014.

| Search | Query | Items found |
| --- | --- | --- |
| #5 | #1 AND #4 | 274 |
| #4 | #2 OR #3 | 1172 |
| #3 | ((infrared AND venous)) | 357 |
| #2 | ((infrared AND vein)) | 949 |
| #1 | ((infrared AND low-cost)) | 11046 |
